# Emerin plays a crucial role in nuclear invagination and in the nuclear calcium transient

**DOI:** 10.1038/srep44312

**Published:** 2017-03-14

**Authors:** Masaya Shimojima, Shinsuke Yuasa, Chikaaki Motoda, Gakuto Yozu, Toshihiro Nagai, Shogo Ito, Mark Lachmann, Shin Kashimura, Makoto Takei, Dai Kusumoto, Akira Kunitomi, Nozomi Hayashiji, Tomohisa Seki, Shugo Tohyama, Hisayuki Hashimoto, Masaki Kodaira, Toru Egashira, Kenshi Hayashi, Chiaki Nakanishi, Kenji Sakata, Masakazu Yamagishi, Keiichi Fukuda

**Affiliations:** 1 Department of Cardiology, Keio University School of Medicine, 35 Shinanomachi Shinjuku-ku, Tokyo 160-8582, Japan; 2 Division of Cardiovascular Medicine, Kanazawa University Graduate School of Medicine, Takara-machi 13-1, Kanazawa, Ishikawa 920-8640, Japan; 3 Electron Microscope Laboratory, Keio University School of Medicine, Tokyo 160-8582, Japan

## Abstract

Alteration of the nuclear Ca^2+^ transient is an early event in cardiac remodeling. Regulation of the nuclear Ca^2+^ transient is partly independent of the cytosolic Ca^2+^ transient in cardiomyocytes. One nuclear membrane protein, emerin, is encoded by EMD, and an EMD mutation causes Emery-Dreifuss muscular dystrophy (EDMD). It remains unclear whether emerin is involved in nuclear Ca^2+^ homeostasis. The aim of this study is to elucidate the role of emerin in rat cardiomyocytes by means of hypertrophic stimuli and in EDMD induced pluripotent stem (iPS) cell-derived cardiomyocytes in terms of nuclear structure and the Ca^2+^ transient. The cardiac hypertrophic stimuli increased the nuclear area, decreased nuclear invagination, and increased the half-decay time of the nuclear Ca^2+^ transient in cardiomyocytes. Emd knockdown cardiomyocytes showed similar properties after hypertrophic stimuli. The EDMD-iPS cell-derived cardiomyocytes showed increased nuclear area, decreased nuclear invagination, and increased half-decay time of the nuclear Ca^2+^ transient. An autopsied heart from a patient with EDMD also showed increased nuclear area and decreased nuclear invagination. These data suggest that Emerin plays a crucial role in nuclear structure and in the nuclear Ca^2+^ transient. Thus, emerin and the nuclear Ca^2+^ transient are possible therapeutic targets in heart failure and EDMD.

Intracellular Ca^2+^ plays central roles in physiology and pathophysiology of cardiomyocytes, such as excitation-contraction (EC) coupling and secondary messengers^1^,^2^. Homeostatic Ca^2+^ oscillation directly regulates rhythmical cardiomyocyte contractions. During a cardiomyocyte contraction, cardiac myocyte membrane depolarization causes the voltage-dependent L-type Ca^2+^ channel (LTCC) to drive Ca^2+^ entry into the cytoplasm^3^. The increase in intracellular Ca^2+^ concentration triggers a greater release of Ca^2+^ from the sarcoplasmic reticulum (SR). The excess Ca^2+^ binds to the myofilament and induces the subsequent myocyte contraction. During cardiomyocyte relaxation, cytoplasmic Ca^2+^ is exported outside the cell via the sarcolemmal Na^+^/Ca^2+^ exchanger (NCX) and pumped into SR through the SR Ca^2+^ ATPase (SERCA). In addition to the role in contraction, intracellular Ca^2+^ has a definitive function as a secondary messenger for various cellular physiological processes. The increased Ca^2+^ level stimulates Ca^2+^-calmodulin (CaM)-dependent protein phosphatase 2B (calcineurin), which activates nuclear factor of activated T cells (NFAT) to drive transcription of several genes^4^. The cytoplasmic Ca^2+^ signal also activates calmodulin-dependent kinases (CaMKs), which positively regulate several transcription factors including myocyte-enhancing factor 2 s (MEF2s) and mediate nuclear translocation of chromatin-modifying enzymes including histone deacetylases (HDACs)^5^,^6^. There are many signaling pathways mediated by cytoplasmic Ca^2+^ in cardiomyocytes; these pathways are intimately involved in development, physiology, and pathophysiology of the heart. Nonetheless, it is unclear how oscillations in Ca^2+^ regulate signaling pathways, e.g., peak Ca^2+^, diastolic Ca^2+^, and micro location^7^. Among several organelles within cardiomyocytes, the nucleus is an exclusive location for transcription and is separated from the cytoplasm by the nuclear envelope. Ca^2+^ oscillation in the nucleus (just as in the cytoplasm) is also observed in cardiomyocytes^8^. The regulation of the so-called nuclear Ca^2+^ transient is partly independent of the cytoplasmic Ca^2+^ transient in cardiomyocytes^9^. The perinuclear microdomains are believed to control the nuclear Ca^2+^ transient^10^,^11^. Moreover, alteration of the nuclear Ca^2+^ transient is an early event in cardiac remodeling^12^. These data suggest that the nuclear Ca^2+^ transient is finely regulated and plays an important role in cardiac pathophysiology, but it remains largely unclear how the nuclear Ca^2+^ transient is regulated in cardiomyocytes.

To understand the molecular mechanism of nuclear Ca^2+^ regulation, we focused on a specific molecule among the nuclear membrane proteins. We also focused on a genetic disease that is caused by a mutation in a nuclear membrane-associated gene and has a specific cardiac phenotype. Emerin (encoded by EMD) is ubiquitously expressed and located in the inner nuclear membrane^13^. A mutation in EMD in humans is a cause of Emery-Dreifuss muscular dystrophy (EDMD) and results in a disorder of skeletal muscle and cardiac muscle including a cardiac conduction aberration and dilated cardiomyopathy (DCM)^14^,^15^,^16^. Emerin binds to several nuclear proteins and performs various functions in the nuclear lamina, nuclear structure, transcriptional regulation, and chromatin architecture^17^,^18^,^19^. It is still largely uncertain how EDMD can be caused by the mutation in EMD. In particular, it is interesting how the mutation in the ubiquitous gene EMD causes a specific phenotype in heart and skeletal muscles. Ca^2+^ signaling is highly important for homeostasis within a contracting muscle, e.g., skeletal myocytes and cardiac myocytes. Disruption of Ca^2+^ signaling in cardiomyocytes results in cardiac hypertrophy and heart failure^20^,^21^. It remains unclear whether a nuclear membrane protein is involved in the regulation of the nuclear Ca^2+^ transient. Therefore, we hypothesized that emerin is involved in nuclear Ca^2+^ signaling in cardiomyocytes and participates in the pathogenesis of some cardiac diseases.

We analyzed nuclear structure and the nuclear Ca^2+^ transient in neonatal rat ventricular cardiomyocytes (NRVCs) after hypertrophic stimuli and in EDMD-induced pluripotent stem (iPS) cell-derived cardiomyocytes. The hypertrophic stimuli increased the nuclear area, decreased nuclear invagination, and increased the half-decay time of the nuclear Ca^2+^ transient in NRVCs. An Emd knockdown also increased the nuclear area, decreased the nuclear invagination, and increased the half-decay time of the nuclear Ca^2+^ transient without hypertrophic stimuli in NRVCs. Hypertrophic-stimulus-dependent changes in nuclear invagination and Ca^2+^ transients were attenuated by the Emd knockdown. The iPS cells were derived from a patient with EDMD and were induced to differentiate into cardiomyocytes. EDMD-iPS cell-derived cardiomyocytes also showed increased nuclear area, decreased nuclear invagination, and increased half-decay time of the nuclear Ca^2+^ transient. Furthermore, an autopsied heart from a patient with EDMD showed an increase in nuclear area and a decrease in nuclear invagination. These data suggest that emerin has a crucial function in nuclear structure and in the nuclear Ca^2+^ transient in cardiomyocytes. Thus, emerin and the nuclear Ca^2+^ transient are possible therapeutic targets in heart failure and EDMD.

## Results

### Hypertrophic stimuli affect nuclear size and invagination in cardiomyocytes

**Figure 1:**
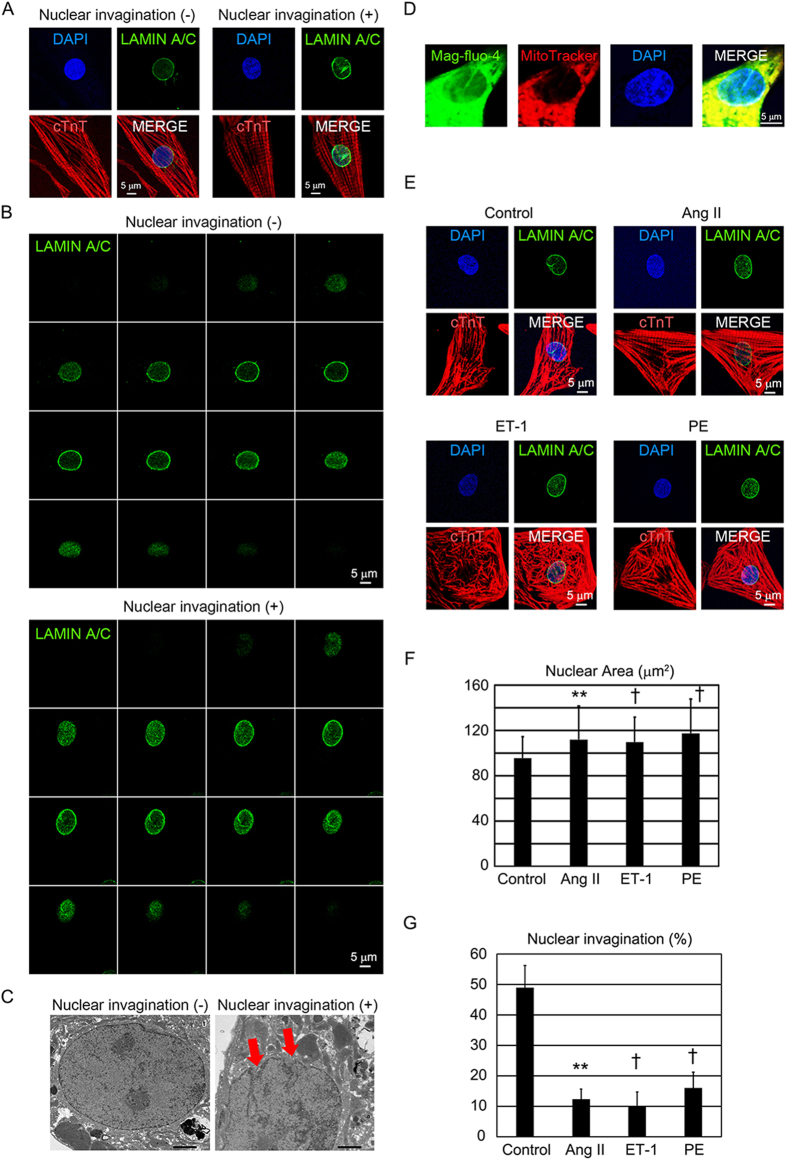
Nuclear structure with hypertrophic stimuli in neonatal rat ventricular cardiomyocytes (NRVCs). (**A**) Confocal microscopic images of immunostaining for cTnT (red), LAMIN A/C (green), and nuclei (blue) in NRVCs. The scale bar is 5 μm. Left: a cardiomyocyte without nuclear invagination. Right: a cardiomyocyte with nuclear invagination. (**B**) Confocal microscopic images of immunostaining for LAMIN A/C (green) with 3D reconstruction, in NRVCs. The scale bar is 5 μm. Top: a cardiomyocyte without nuclear invagination. Bottom: a cardiomyocyte with nuclear invagination. (**C**) Electron microscopic images of NRVC. The scale bar is 2 μm. Left: a cardiomyocyte without nuclear invagination. Right: a cardiomyocyte with nuclear invagination. Red arrow indicates nuclear invagination. (**D**) Confocal microscopic images of Mag-fluo-4 fluorescence (green), MitoTracker (red), and nuclei (blue). The scale bar is 5 μm. (**E**) Representative confocal microscopic images of immunostaining for cTnT (red), LAMIN A/C (green), and nuclei (blue) in control NRVCs and NRVCs subjected to hypertrophic stimuli: Ang II, ET-1, or PE. The scale bar is 5 μm. (**F**) Bar graphs showing nuclear size in NRVCs exposed to vehicle (control; n = 100), Ang II (n = 26), ET-1 (n = 108), or PE (n = 79) for 48 hours in culture. The data are mean ± SD. (**G**) Bar graphs showing the percentages of cardiomyocytes with nuclear invagination among NRVCs exposed to vehicle (control; n = 47), Ang II (n = 97), ET-1 (n = 40), or PE (n = 56) for 48 hours in culture. *P < 0.05 compared to the control. **P < 0.01 compared to the control. †P < 0.001 compared to the control.

Nuclear size and shape are important for cellular fine structure and function. Altered nuclear size and shape are associated with a wide variety of pathologies^22^,^23^. The number of nuclear envelop intrusions into the nuclear lumen (nuclear invagination) in cardiomyocytes is decreased during cardiac remodeling^12^. To study the nuclear structure in cardiomyocytes, we examined nuclear structure by immunofluorescence staining in NRVCs. We observed NRVCs with or without nuclear invagination in the basal condition (Fig. 1A, Supplementary Figure 1A,B). We also observed nuclear invagination by 3D reconstruction image and electron microscopy (Fig. 1B,C, Supplementary Figure 1C). The low-affinity Ca^2+^ indicator mag-fluo-4 can reveal the nuclear envelop and tubular invaginations that traverse the nucleus, which imply that the nuclear envelop and its tubular invaginations are functional Ca^2+^ stores. MitoTracker Red specifically accumulated in both the subsarcomeric mitochondria (located around the nucleus) and in the intermyofibrillar mitochondria, and the fluorescence intensity of cardiomyocytes is known to be much stronger than that of nonmyocytes^24^. The mag-fluo-4 staining showed the nuclear invagination of the NRVCs in the basal condition (Fig. 1D). Next, we examined the effects of each hypertrophic stimulus on the nuclear size and shape in NRVCs. We used potent hypertrophic stimuli, such as angiotensin II (Ang II), endothelin 1 (ET-1), and phenylephrine (PE)^25^ (Fig. 1E, Supplementary Figure 1D). Given the prominent role of these cytokines as strong hypertrophy-promoting factors, we initially confirmed the cardiomyocyte hypertrophy by measuring the cell surface area (Supplementary Figure 1E), and then we examined the nuclei. The nuclear size was significantly increased by exposure to these hypertrophic stimuli (Fig. 1F, Supplementary Figure 1F,G). Nuclear invagination was defined as a nuclear intrusion consist of two membranes continuous with nuclear envelope observed by immunostaining for nuclear membrane proteins^26^,^27^, and nuclear tubular structures longer than 1 μm^12^. In this study, we defined nuclear invaginations as tubular structures continuous with nuclear envelope longer than 1 μm by immunostaining for nuclear membrane proteins. The incidence of cardiomyocytes with a nuclear invagination was significantly decreased by exposure to the hypertrophic stimuli (Fig. 1G, Supplementary Figure 1H).

### Hypertrophic stimuli affect the nuclear Ca^2+^ transient

**Figure 2:**
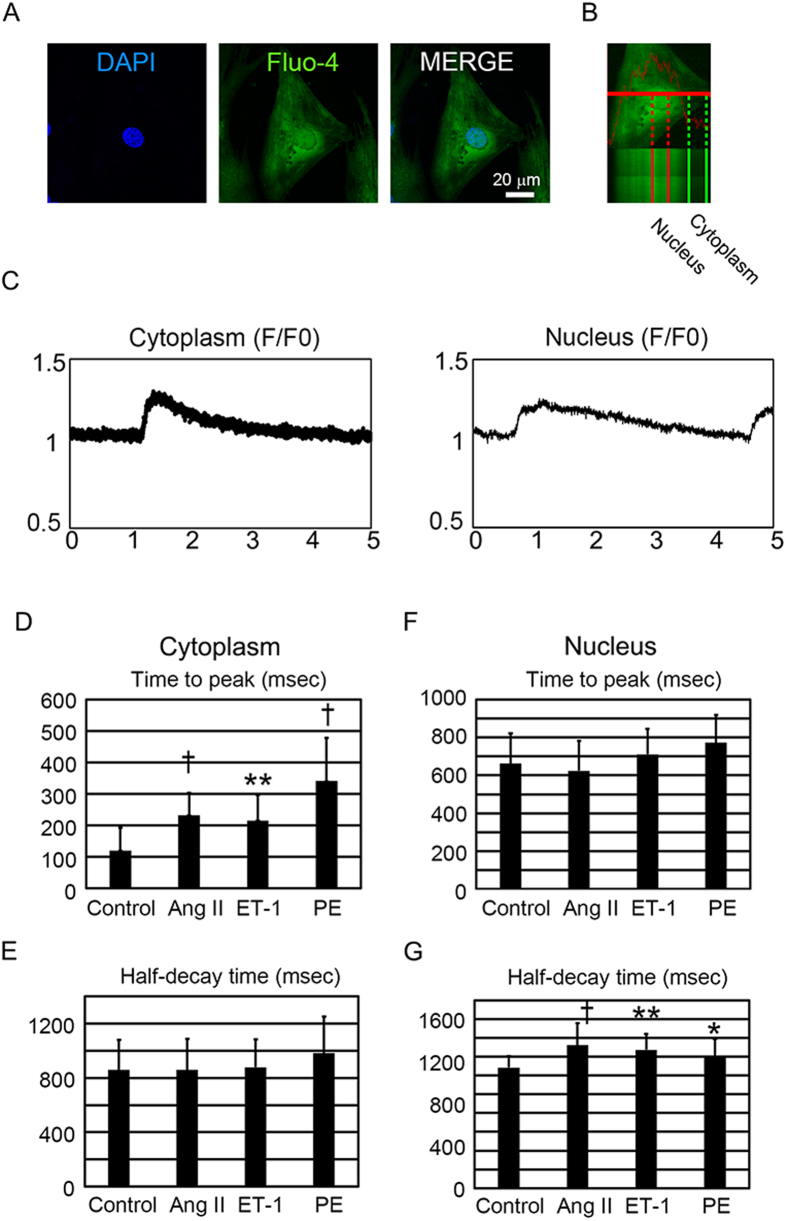
The nuclear Ca^2+^ transient in neonatal rat ventricular cardiomyocytes (NRVCs) subjected to hypertrophic stimuli. (**A**) Confocal microscopic images of Fluo-4 fluorescence (green) and nuclei (blue). The scale bar is 20 μm. (**B**) Characterization of cytoplasmic and nuclear Ca^2+^ transients in NRVCs. Line scan imaging of cytoplasmic (Left) and nuclear (Right) Ca^2+^ transients in cardiomyocytes. (**C**) Original recordings of nuclear and cytoplasmic Ca^2+^ transients of control NRVC. (**D**,**E**) Average values of the effects of hypertrophic stimuli on time to peak and half-decay time in the cytoplasmic of NRVCs exposed to vehicle (control; n = 19), Ang II (n = 42), ET-1 (n = 21), or PE (n = 29). (**F**,**G**) Average values of the effects of hypertrophic stimuli on time to peak and half-decay time in the nuclei of NRVCs exposed to vehicle (control; n = 19), Ang II (n = 42), ET-1 (n = 21), or PE (n = 29). *P < 0.05 compared to the control. **P < 0.01 compared to the control. ^†^P < 0.001 compared to the control.

Although the cytosolic Ca^2+^ transient plays important roles in the pathogenesis of heart diseases, it is mostly unclear whether the nuclear Ca^2+^ transient in cardiomyocytes would be affected by various hypertrophic stimuli. To analyze the kinetics of nuclear and cytoplasmic Ca^2+^ transients, NRVCs were loaded with the Ca^2+^ indicator Fluo-4, which diffuses both to the cytoplasm and nucleus, and rapid resolution confocal microscopic analysis was then conducted^28^,^29^. Fluo-4 staining discriminated the area of the nucleus (Fig. 2A), and line scan imaging was performed both in the cytoplasm and nucleus within the same cardiomyocytes (Fig. 2B,C, Supplementary Figure 2A,B,C,D). We also used different type of Ca^2+^ indicator, Rhod4 which had different Kd from fluo4, and observed similar nuclear Ca^2+^ dynamics (Supplementary Figure 2E,F,G). Time to peak in cytoplasmic Ca^2+^ transient was significantly increased by hypertrophic stimuli (Fig. 2D), but time to peak in nuclear Ca^2+^ transient was not affected by these stimuli (Fig. 2F). Half-decay time of cytoplasmic Ca^2+^ transient was not affected by hypertrophic stimuli (Fig. 2E), but that of nucleus was significantly increased by hypertrophic stimuli (Fig. 2G). F/F0 in cytoplasmic and nuclear Ca^2+^ transient was not uniformly affected by hypertrophic stimuli (Supplementary Figure 2H,I). These data indicate that the effect of hypertrophic stimuli on the nuclear Ca^2+^ transient is independent of the cytoplasmic Ca^2+^ transient.

### Emerin is involved in nuclear structure and in the Ca^2+^ transient in neonatal rat ventricular cardiomyocytes

**Figure 3:**
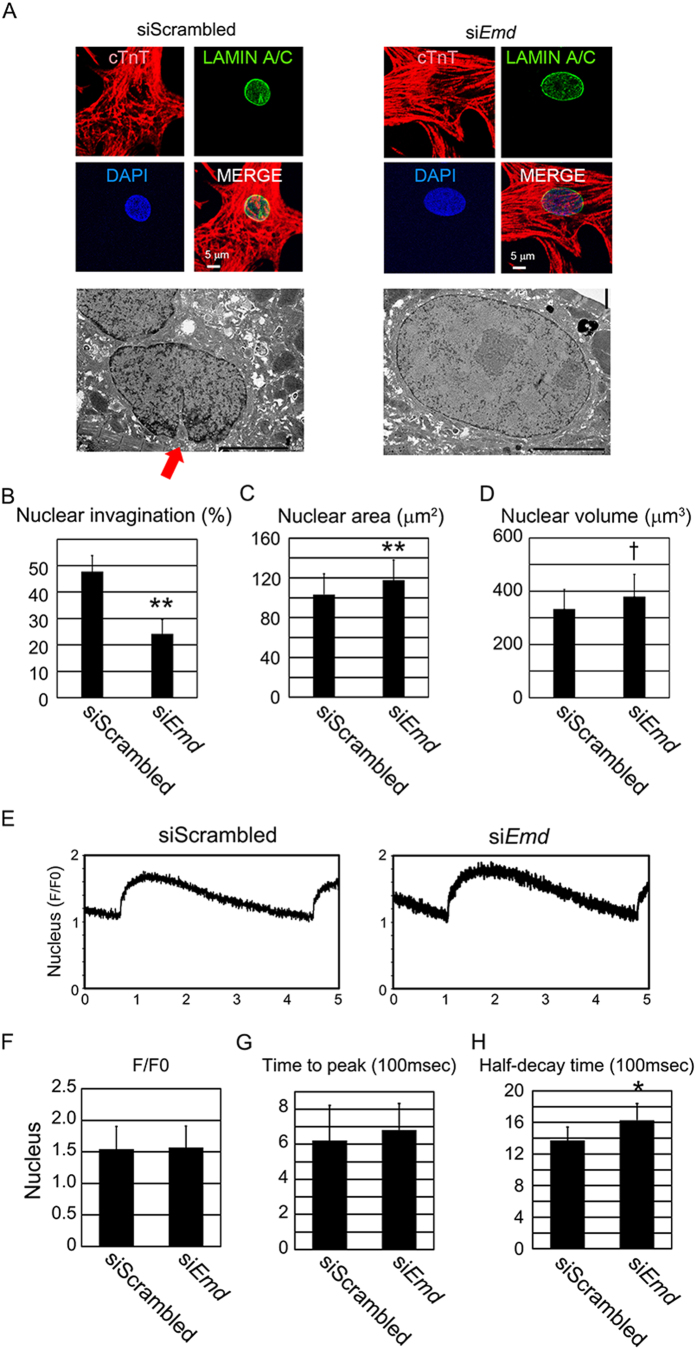
Nuclear structure and the nuclear Ca^2+^ transient in Emd knockdown neonatal rat ventricular cardiomyocytes (NRVCs). (**A**) Top: Confocal microscopic images of immunostaining for cTnT (red), LAMIN A/C (green) and nuclei (blue) in NRVCs with control scrambled siRNA (siScrambled, left) and siRNA against Emd (siEmd, right). Bottom: Electron microscopic images of NRVCs with control scrambled siRNA (siScrambled, left) and siRNA against Emd (siEmd, right). The scale bar is 5 μm. Red arrow indicates nuclear invagination. (**B**) Bar graphs showing the percentages of cardiomyocytes with nuclear invagination in NRVCs exposed to siScrambled (n = 69) and siEmd (n = 62) for 48 hours in culture. The data are mean ± SE. (**C**) Bar graphs showing the percentages of cardiomyocytes with nuclear size in NRVCs exposed to siScrambled (n = 69) and siEmd (n = 62) for 48 hours in culture. The data are mean ± SD. (**D**) Bar graph showing nuclear volumes in NRVCs exposed to siScrambled (n = 95) or siEmd (n = 138). The data are mean ± SD. (**E**) Original recordings of the nuclear Ca^2+^ transient of NRVCs with siScrambled (n = 25) and siEmd (n = 29). (**F**,**G**,**H**) Average values of the effects of the Emd knockdown on F/F0, time to peak, and half-decay time in the nuclei of NRVCs. *P < 0.05 compared to the control. **P < 0.01 compared to the control. †P < 0.001 compared to the control.

To examine the role of emerin in cardiomyocytes, the cellular and nuclear structures were studied by immunofluorescent staining and electron microscopy in NRVCs during an siRNA-mediated Emd knockdown (Fig. 3A,B,C). SiRNA against Emd in cardiomyocytes efficiently downregulated the expression of Emd (Supplementary Figure 3A). Although cell surface area was not changed by siRNA against Emd (Supplementary Figure 3B), the incidence of cardiomyocytes with nuclear invagination was significantly decreased (Fig. 3B), and the nuclear size was significantly increased by transfection with siRNA against Emd (Fig. 3C,D, Supplementary Figure 3C). These data suggest that emerin has a pivotal function in the maintenance of nuclear invagination and nuclear size. Nuclear structural changes are associated with altered nuclear Ca^2+^ regulation in cardiomyocytes^12^. Then, we analyzed the Ca^2+^ transient both in the cytoplasm and nucleus in NRVCs during the Emd knockdown (Supplementary Figure 3E, Fig. 3E). F/F0 and time to peak were not affected in cytoplasmic Ca^2+^ and nuclear Ca^2+^ by siRNA against Emd (Fig. 3F,G, Supplementary Figure 3F,G,H). Half-decay time of the cytoplasmic Ca^2+^ was not affected by siRNA against Emd (Supplementary Figure 3F,H) but that of the nuclear Ca^2+^ was significantly increased by siRNA for Emd (Fig. 3H, Supplementary Figure 3G). These data suggest that emerin plays a specific role in the nuclear Ca^2+^ transient, independently of the cytoplasmic Ca^2+^ transient.

### Emerin is indispensable for the hypertrophic-stimulus-induced change in the nuclear Ca^2+^ transient in neonatal rat ventricular cardiomyocytes

**Figure 4:**
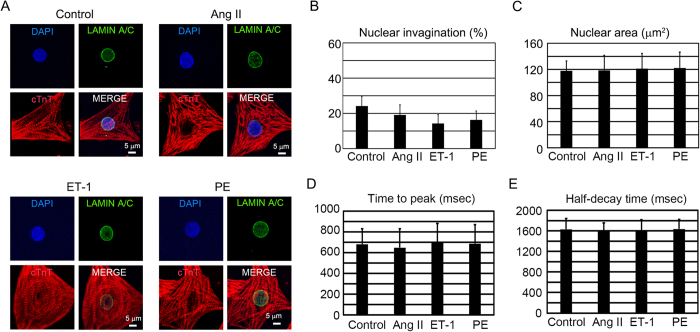
Nuclear structure and the nuclear Ca^2+^ transient in Emd knockdown neonatal rat ventricular cardiomyocytes (NRVCs) subjected to hypertrophic stimuli. (**A**) Confocal microscopic images of immunostaining for cTnT (red), LAMIN A/C (green), and nuclei (blue) in NRVCs treated with siRNA against Emd after exposure to vehicle (control; n = 62), Ang II (n = 47), ET-1 (n = 56), or PE (n = 55) for 48 hours in culture. The scale bar is 20 μm. (**B**) Bar graphs showing the percentages of cardiomyocytes with nuclear invagination in NRVCs treated with siRNA against Emd after exposure to vehicle (control; n = 62), Ang II (n = 47), ET-1 (n = 56), or PE (n = 55) for 48 hours in culture. The data are mean ± SE. (**C**) Bar graphs showing nuclear size of cardiomyocytes with nuclear invagination in NRVCs treated with siRNA against Emd after exposure to vehicle (control; n = 62), Ang II (n = 47), ET-1 (n = 56), or PE (n = 55) for 48 hours in culture. The data are mean ± SD. (**D**,**E**) Average values of the effects of hypertrophic stimuli on time to peak and half-decay time in the nucleus of NRVCs treated with siRNA against Emd after exposure to vehicle (control; n = 29), Ang II (n = 25), ET-1 (n = 49), and PE (n = 24).

To examine the relation between hypertrophic stimuli and emerin in cardiomyocytes, we used hypertrophic stimuli in cardiomyocytes with the Emd knockdown. The cellular and nuclear structures were examined by immunofluorescent staining in NRVCs (Fig. 4A). The hypertrophic stimuli had no significant effect on the nuclear invagination and nuclear area under Emd knockdown (Fig. 4B,C, Supplementary Figure 4A). Ca^2+^ transients of the cytoplasm and nucleus were also examined. Time to peak in cytoplasmic Ca^2+^ transient was significantly increased by the hypertrophic stimuli (Supplementary Figure 4D), but that in nuclear Ca^2+^ transient was not affected by hypertrophic stimuli under Emd knockdown (Fig. 4D). Half-decay times of cytoplasmic and nuclear Ca^2+^ transients were not affected by hypertrophic stimuli under Emd knockdown (Supplementary Figure 4E, Fig. 4E). The increases by hypertrophic stimuli in time to peak of cytoplasmic Ca^2+^ transient under Emd knockdown (Supplementary Figure 4D) was similar with that without Emd knockdown (Fig. 2D). Although hypertrophic stimuli increase half-decay time of nuclear Ca^2+^ transient (Fig. 2G), hypertrophic stimuli did not affect half-decay time of nuclear Ca^2+^ transient under Emd knockdown (Fig. 4E). To examine whether emerin induces nuclear invagination, we examined the nuclear structure under overexpression of emerin in NRVCs (Supplementary Figure 4G,H). Nuclear invagination is significantly increased by emerin overexpression (Supplementary Figure 4I). These results suggest that emerin would play a role in nuclear membrane structure and affect the nuclear Ca^2+^ transient during exposure to hypertrophic stimuli, which would be because nuclear invagination has a critical role in the nuclear Ca^2+^ transient^28^,^29^.

### Recapitulation of the cellular phenotype in EDMD-iPS cell-derived cardiomyocytes

**Figure 5:**
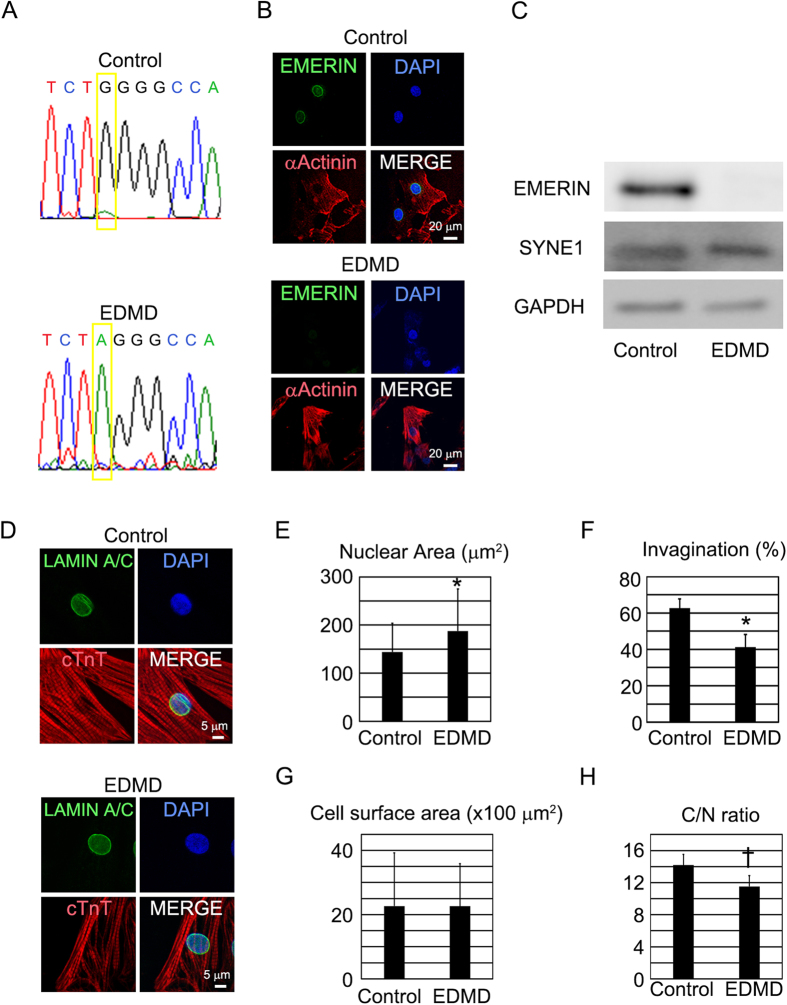
Nuclear structure and the nuclear Ca^2+^ transient in EDMD-iPS cell-derived cardiomyocytes. (**A**) Sequence analysis of genomic EMD in control and EDMD-iPS cells. Single nucleotide substitution G to A at position 1735 of EMD. (**B**) Confocal microscopic images of immunostaining for α-Actinin (red), EMERIN (green), and nuclei (blue) in control and EDMD-iPS cell-derived cardiomyocytes. The scale bar is 20 μm. (**C**) Western blotting for EMERIN, SYNE1, and GAPDH in control and EDMD-iPS cell-derived cardiomyocytes. (**D**) Confocal microscopic images of immunostaining for cTnT (red), LAMIN A/C (green), and nuclei (blue) in control and EDMD-iPS cell-derived cardiomyocytes. The scale bar is 5 μm. (**E**) Bar graphs showing nuclear size in control (n = 94) and EDMD-iPS cell-derived cardiomyocytes (n = 50). The data are mean ± SD. (**F**) Bar graphs showing the percentages of cardiomyocytes with nuclear invagination in control (n = 94) and EDMD-iPS cell-derived cardiomyocytes (n = 50). The data are mean ± SE. (**G**) Bar graphs showing cell surface area in control (n = 94) and EDMD-iPS cell-derived cardiomyocytes (n = 50). The data are mean ± SD. (**H**) Bar graphs showing the ratio of cell surface area to nuclear area in control and EDMD-iPS cell-derived cardiomyocytes. The data are mean ± SD. *P < 0.05 compared to the control. **P < 0.01 compared to the control. ^†^P < 0.001 compared to the control.

We successfully derived iPS cells from a patient with EDMD. As controls, we used iPS cells derived from healthy volunteers: previously characterized control iPS cell lines^30^,^31^,^32^. At first, we confirmed the mutation in EMD by direct sequencing of DNA from EDMD-iPS cells. Sequence analysis of EMD showed a c.1735 G > A mutation (Fig. 5A). One report indicated that this mutation results in a deficiency of emerin expression^33^. The generated EDMD-iPS cells and control iPS cells showed stem cell marker expression (Supplementary Figure 5A). Subsequently, cardiomyocytes were produced from the iPS cells, and immunostaining revealed that these iPS cell-derived cardiomyocytes expressed cardiomyocyte-specific markers: α-Actinin, HCN4, NKX2.5, and cardiac troponin T (cTnT), and showed normal cardiomyocyte cellular structure (Supplementary Figure 5B). In electron-microscopic images of beating cardiomyocytes, we could not find any morphometric aberrations in EDMD-iPS cell-derived cardiomyocytes (Supplementary Figure 5C). We also confirmed that EMERIN is not detected in EDMD-iPS cell-derived cardiomyocytes (Fig. 5B,C). Distribution of nuclear membrane protein, such as LAMIN A/C, SUN1 and SYNE1, which also can be causal mutations in EDMD, were not changed by a deficiency of EMERIN (Fig. 5D, Supplementary Figure 5D,E). Next, we analyzed the morphological features of the nucleus and cytoplasm of iPS cell-derived cardiomyocytes. The nuclear area was significantly increased (Fig. 5E), and the incidence of cardiomyocytes with nuclear invagination was significantly decreased in EDMD-iPS cell-derived cardiomyocytes (Fig. 5F). There were no significant differences in the cell surface area between control and EDMD-iPS cell-derived cardiomyocytes (Fig. 5G,H). These data suggest that EDMD-iPS cell-derived cardiomyocytes decreased nuclear invagination similar to that of NRVCs transfected with siRNA against EMD.

### Emerin is indispensable for the nuclear Ca^2+^ transient in EDMD-iPS cell-derived cardiomyocytes

**Figure 6:**
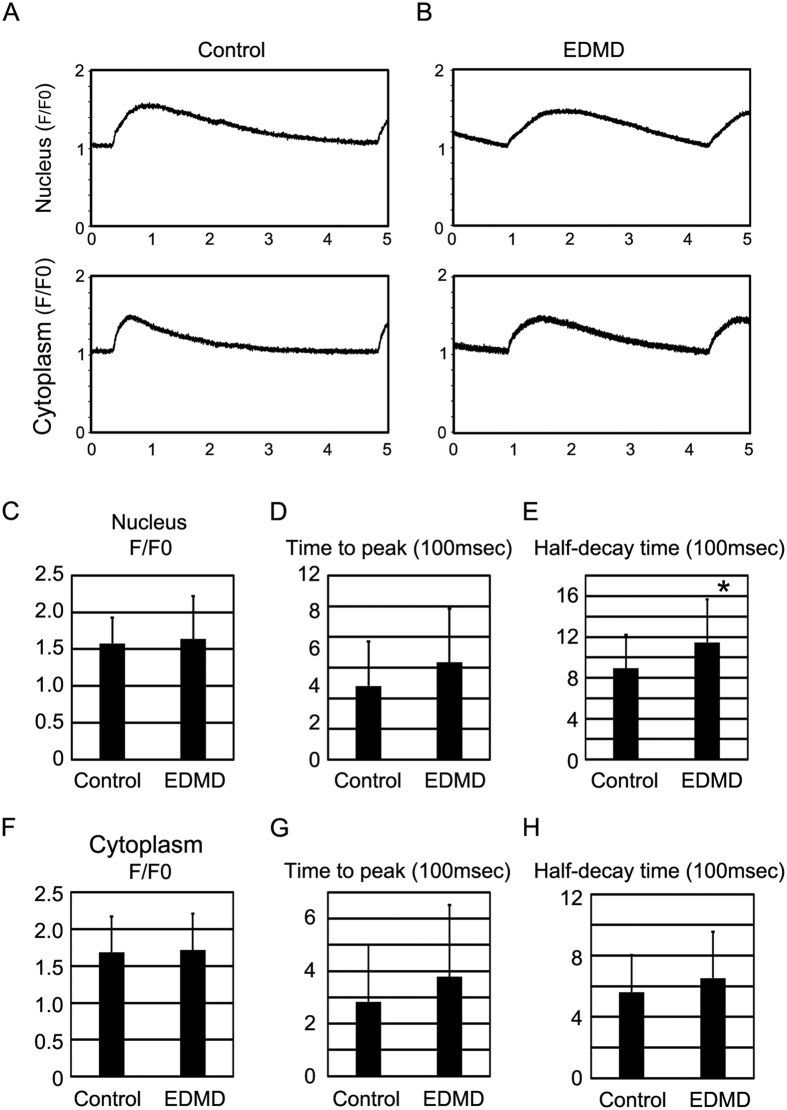
The nuclear Ca^2+^ transient in EDMD-iPS cell-derived cardiomyocytes. (**A**) Original recordings of nuclear and cytoplasmic Ca^2+^ transients of control iPS cell-derived cardiomyocytes. (**B**) Original recordings of nuclear and cytosolic Ca^2+^ transient of EDMD-iPS cell-derived cardiomyocytes. (**C**,**D**,**E**) Average values of F/F0, time to peak, and half-decay time in the nuclear Ca^2+^ transient of control (n = 16) and EDMD-iPS cell-derived cardiomyocytes (n = 25). (**F**,**G**,**H**) Average values of F/F0, time to peak, and half-decay time in the cytoplasmic Ca^2+^ transient of control (n = 16) and EDMD-iPS cell-derived cardiomyocytes (n = 25). *P < 0.05 compared to the control.

Ca^2+^ transients of cytoplasm and nucleus were also examined in control and EDMD-iPS cell-derived cardiomyocytes (Fig. 6A,B). There were no significant differences in cytoplasmic Ca^2+^ transient and nuclear Ca^2+^ transient in F/F0 and time to peak between control and EDMD-iPS cell-derived cardiomyocytes (Fig. 6C,D,F,G). Half decay time of nuclear Ca^2+^ transient was increased in EDMD-iPS cell-derived cardiomyocytes, but there was no significant change in half-decay time of cytoplasmic Ca^2+^ transient (Fig. 6E,H). These data suggest that EDMD-iPS cell-derived cardiomyocytes have Ca^2+^ transient similar to that of NRVCs treated with siRNA against EMD and indicate that emerin performs a critical function in the nuclear Ca^2+^ transient in human cardiomyocytes.

### Cardiac nuclear morphology is disrupted in the heart of a patient with EDMD

**Figure 7:**
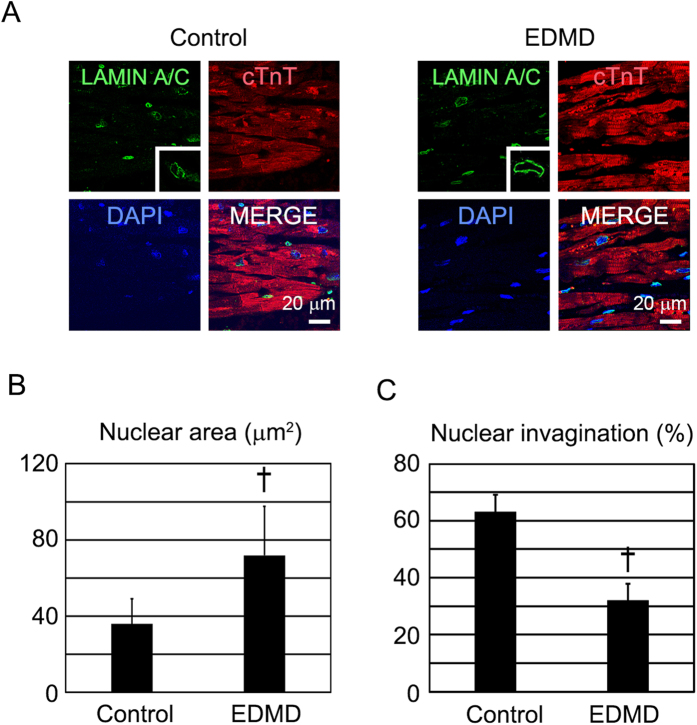
Cardiac nuclear morphology in an autopsied heart of a patient with EDMD. (**A**) Confocal microscopic images of immunostaining for cTnT (red), emerin (green), and nuclei (blue) in control and EDMD-heart. The scale bar is 20 μm. (**B**) Bar graphs showing nuclear size in control and EDMD-heart (n = 65). The data are mean ± SD. (**C**) Bar graphs showing the percentages of cardiomyocytes with nuclear invagination in control and EDMD-heart (n = 65). The data are mean ± SE. *P < 0.05 compared to the contro*l*.

To study the diseased adult heart, we used an autopsied heart from a patient with EDMD (EDMD-heart). We performed immunostaining for LAMIN A/C to elucidate the nuclear morphology and immunostaining for cTnT to identify the cardiomyocytes (Fig. 7A). The nuclear area was significantly increased in the EDMD-heart (Fig. 7B). The incidence of cardiomyocytes with nuclear invagination was significantly decreased in the EDMD-heart (Fig. 7C). These data suggest that the heart from an adult patient with EDMD has nuclear structure similar to that of NRVCs treated with siRNA and of EDMD-iPS cell derived cardiomyocytes.

## Discussion

Cytoplasmic Ca^2+^ has several physiological functions in cardiomyocytes. Cardiac contraction is finely regulated by cytoplasmic Ca^2+^: EC coupling. Depolarization of the plasma membrane in cardiomyocytes activates LTCC to open in order to increase Ca^2+^ influx into the cytoplasm, but intracellular Ca^2+^-dependent inactivation limits Ca^2+^ influx during an action potential^34^. This locally upregulated Ca^2+^ binds to the ryanodine receptor (RyR) in SR adjacent to the plasma membrane, and this event triggers a massive efflux of Ca^2+^ from the SR into the cytoplasm: Ca^2+^-induced Ca^2+^ release (CICR)^1^. During the relaxation of cardiomyocytes, Ca^2+^ is sequentially removed from the cytoplasm. It seems that cytoplasmic Ca^2+^ is synchronously changed in the whole cytoplasm, but cytoplasmic Ca^2+^ is finely and locally regulated with respect to each microcompartment within the cell.

The nuclear Ca^2+^ transient shows different kinetics from those of the cytoplasmic Ca^2+^ transient^35^ and has its own role in target gene expression, independent of cytoplasmic Ca^2+^ 36,37. The nuclear envelope (NE), which is continuous with the endoplasmic reticulum (ER), not only contributes to nuclear structure and insulation from the surrounding cytoplasm but also stores Ca^2+^ around the nucleus^38^. The NE generates Ca^2+^ signals independently of cytoplasmic Ca^2+^ 39. The NE is composed of two membranes: the outer nuclear membrane (ONM), a structure continuous with ER, and the inner nuclear membrane (INM) exposed to the nuclear matrix. The ONM harbors SERCA2, which actively transports Ca^2+^ from the cytoplasm surrounding the nucleus into the NE^40^. There are also the NCX complex and inositol trisphosphate (IP3) receptor in the INM, which are thought to be responsible for Ca^2+^ homeostasis in the nucleoplasm^28^,^29^. Furthermore, nuclear Ca^2+^ is also controlled by several nuclear membrane proteins, such as RyRs and nicotinic acid adenine dinucleotide phosphate receptors (NAADPRs)^41^,^42^.

The cellular environment affects the nuclear envelop shape including nuclear invagination^43^. These nuclear structures participate in cellular functions including nuclear Ca^2+^ signaling^44^. Nuclear invagination is required for prompt Ca^2+^ pump back to cytoplasm^12^,^45^,^46^, like T tubule in cytoplasmic Ca^2+^. In cardiomyocytes, nuclear invagination is firstly changed during cardiac remodeling, which suggests that nuclear invagination may play a critical role in hypertrophic gene program activation and cardiac remodeling^12^. It is still unclear how nuclear invagination is regulated, what molecules are involved in the regulation of this process. EMERIN is a nuclear membrane protein and binds to SUN1, SUN2 and SYNE1, which connect cytoplasm to nucleus and play a role in nuclear fine structure^19^,^47^. The decrease in EMERIN would affect those protein interactions and structural maintenance of nuclear invagination. We observed the prolongation of half-decay time but not time to peak, which suggests that the decrease of EMERIN would cause the dysregulation of Ca^2+^ pump back function mediated by nuclear invagination, rather than the dysregulation of Ca^2+^ release mechanism such as IP3 receptors and RyRs. In our experiments, the decrease in nuclear invagination may disturb the prompt Ca^2+^ pump back and prolong the half-decay time. The intracellular Ca^2+^ dynamics activates several transcription factors and co-factors, such as HDAC5, NFAT and MEF2C, which induce cardiac hypertrophy^46^,^48^,^49^. Alteration of the nuclear Ca^2+^ transient is an early event in cardiac remodeling^12^. Before the change in cytoplasmic Ca^2+^ transient, alteration of the nuclear Ca^2+^ transient and HDAC5 activation were observed^12^. The decrease in the half-decay time of nuclear Ca^2+^ which we observed would indicate the increased nuclear Ca^2+^ for a prolonged time in diastolic phase. The increased nuclear Ca^2+^ would activate Ca^2+^ dependent-transcription factors and co-factors in nucleus.

Numerous proteins have been identified in the NE membrane, including both ubiquitous and potentially cell type-specific proteins^50^. Each NE membrane protein has a unique role in spatial control of the genome, cell cycle regulation, or cytoskeletal organization. The precise molecular functions of most NE membrane proteins remain elusive^19^. The mutations known to be responsible for human genetic diseases give us a key to decipher the molecular function and the related disease mechanism. EMD is ubiquitously expressed and encodes a nuclear membrane protein^13^,^51^, but the disease signs in EDMD are restricted to skeletal muscle and cardiac muscle; this situation suggests that emerin may have a specific role in skeletal muscle and cardiac muscle. Here, we reported for the first time that emerin plays a role in nuclear invagination and nuclear Ca^2+^ transient. EDMD is also caused by gene mutations in other nuclear-membrane-associated proteins, such as LMNA, SYNE1, SYNE2, SUN1, SUN2, and TMEM43^52^,^53^,^54^,^55^. Although it remains unclear how emerin regulates nuclear invagination, we can theorize that emerin regulates nuclear invagination in collaboration with the above-mentioned nuclear membrane proteins. We showed that an EDMD patient’s cardiomyocytes have a defect in nuclear invagination, but it is still not clear whether human adult cardiomyocytes have a defect in nuclear Ca^2+^ transients and what happens next to the defect of the nuclear Ca^2+^ transient. These issues should be clarified in the future. We also observed the increase of nuclear size. Nuclear size is regulated by several mechanisms such as nucleocytoplasmic transport, nuclear structural components, and post-mitotic nuclear assembly^56^. Nuclear architecture directly affects chromatin organization and regulates gene expression. Some factors that structure and modify chromatin also influence nuclear morphology and size^56^. Nuclear size would also possibly affect chromatin organization and gene expression. Previous report showed that hypertrophic stimuli induced the increase of nuclear size^57^. We speculate that the change in nuclear size by hypertrophic stimuli might play a role in gene expression related to cardiac hypertrophy.

Human cardiomyocytes from patients with genetic disorders are an ideal tool for clinical research, but their use is limited in basic research. Most basic researches conduct studies using animal models for research into relevant diseases. It should be noted that there are many differences between animals and humans, and some human diseases cannot be reproduced in animal models. Here, we used iPS cell-derived cardiomyocytes to elucidate the cellular pathophysiology of the patient with EDMD. Recent methodological and technological progress can help researchers to derive iPS cells from patients with genetic disorders, induce iPS cells to differentiate into cardiomyocytes, and then to purify the cardiomyocytes^58^,^59^,^60^,^61^,^62^,^63^,^64^, but such studies still require much labor and funding. Patient-specific iPS cells have contributed to disease modeling and drug screening^30^,^31^,^32^,^65^. Nonetheless, research is still in progress on how to make iPS cell-derived cardiomyocytes mature like adult rod-shaped cardiomyocytes. Therefore, the studies involving patient-specific iPS cells cannot be applied to all genetic diseases.

In summary, nuclear invagination is attenuated and the nuclear Ca^2+^ transient is affected by hypertrophic stimuli in NRVCs. The nuclei of Emd knockdown NRVCs also show characteristics similar to those of hypertrophic-factor-stimulated cardiomyocytes, but cell size and the cytoplasmic Ca^2+^ transient are not affected by the Emd knockdown. Human EDMD-iPS cell-derived cardiomyocytes also show attenuated nuclear invagination and an altered nuclear Ca^2+^ transient. These data suggest that EDMD would be caused by disturbances of nuclear invagination and of the nuclear Ca^2+^ transient. Nuclear invagination and the nuclear Ca^2+^ transient may be therapeutic targets in EDMD and cardiac remodeling. In addition, emerin may be a molecular mediator of nuclear invagination and of the nuclear Ca^2+^ transient in the heart.

## Methods

### Rat cardiomyocyte culture

Primary cultures of NRVCs were prepared as described previously^66^. In brief, NRVCs from 1- to 2-day-old Sprague–Dawley rats (CREA, Japan) were killed by decapitation and the hearts were immediately excised and subjected to Percoll gradient centrifugation and differential plating to enrich the cardiac myocyte population and to deplete nonmyocytes. Cardiomyocytes were cultured in a mixture of Dulbecco’s modified Eagle’s medium (DMEM) and M199 (Sigma-Aldrich) with 10% of horse serum (GIBCO) and antibiotic-antimycotic agents (GIBCO). All procedures in the present study conformed to the principles outlined in the Guide for the Care and Use of Laboratory Animals published by the US National Institutes of Health (NIH Publication No. 85-23, revised 1996 and updated 2011) and were approved by the Institutional Animal Care and Use Committee of Keio University School of Medicine.

### Ca^2+^ imaging

Cardiomyocytes were placed in 35-mm glass-bottom dishes and loaded with 5 μM Fluo-4 (Invitrogen) or Rhod4 (AAT bioquest) in Tyrode’s solution (pH 7.40, NaCl 135 mM, NaHPO_4_ 0.33 mM, MgCl_2_∙6H_2_0 0.53 mM, CaCl2∙2H_2_0 1.8 mM, HEPES-NaOH 5 mM, Glucose 5.5 mM) for 30 min at 37 °C. A further 30 min was allowed for deesterification. Ca^2+^ imaging was performed at room temperature by confocal microscopy. Ca^2+^ imaging was performed by confocal microscopy (LSM 5 DUO Carl Zeiss, Jena, Germany) using a ×40/0.75 NA. The pinhole was set to 1 Airy unit, resulting in optical-slice thickness of 1.0 μm. The confocal plane was set to the middle (z-axis) of the nuclei. A 512-pixel scan line was positioned to include the nucleus, and scanning was performed every 2 ms (a total of 10,000 times for a 20-second recoding). Cardiomyocytes spontaneously beating and electrically stimulated at 0.25 Hz were analyzed for F/F_0_, time to peak, and half-decay time.

### Drug testing

Angiotensin II (Sigma-Aldrich), endothelin 1 (Sigma-Aldrich), and phenylephrine (Sigma-Aldrich) were used for the drug assays.

### Small interfering RNA (siRNA)

Rat Emd siRNA (s129800) and scrambled siRNA (4390846) were purchased from Life Technologies. SiRNA was diluted with OPTI-MEM (Invitrogen) to 20 nmol/L and added in the amount of 125 μL to 7.5 μL OPTI-MEM-containing LipofectamineRNAi Max (Invitrogen). After incubation for 10 min at room temperature, the siRNA-Lipofectamine mixture was added to NRVCs with subsequent incubated for 24 hours at 37 °C without horse serum and antibiotic-antimycotic agents. The cells were transferred to a maintenance medium without the horse serum and antibiotic-antimycotic agents, and then 100 nmol/L ET-1, 100 nmol/L Ang II, or 50 μmol/L phenylephrine were added with subsequent incubation for 48 hours. The cells were then harvested for RNA analysis, immunofluorescence staining, and Ca^2+^ imaging.

### Emerin overexpression in NRVC

pCAG-GFP-IRES-Emerin plasmid was constructed by emerin cDNA and pCAG-GFP (Addgene) plasmid. Each 2.5 μg plasmid were diluted with 150 μl OPTI-MEM (Invitrogen) and added in the amount of 125 μL to 3.5 μL OPTI-MEM-containing Lipofectamine3000 (Invitrogen). After incubation for 10 min at room temperature, the mixture was added to NRVCs with subsequent incubated for 48 hours at 37 °C without horse serum and antibiotic-antimycotic agents. The cells were transferred to a maintenance medium without the horse serum and antibiotic-antimycotic agents, and then 50 μmol/L phenylephrine were added with subsequent incubation for 48 hours. The cells were then harvested for immunofluorescence staining.

### Quantitative reverse-transcription (RT) PCR

For quantitative RT-PCR assays, total RNA was extracted from NRVCs (after 4 days of culturing) by means of the TRIzol Reagent (Invitrogen). One microgram of total RNA was reverse-transcribed into cDNA with the ReverTra Ace^®^ qPCR RT Master Mix with gDNA Remover (TOYOBO). Quantitative RT-PCR for emerin (EMD) and GAPDH genes was conducted by means of the Power SYBR^®^ Green PCR Master Mix (Applied Biosystems). Quantitative RT-PCR for EMD and GAPDH was performed with the primer set shown in the Supplementary Table on a ViiA™ 7 Real Time PCR System (Applied Biosystems), and the cycling conditions were as follows: 20 seconds at 95 °C, followed by 40 cycles of 1 second at 95 °C and 20 seconds at 62 °C. Cycle threshold was calculated under default settings by means of real-time sequence detection software (Applied Biosystems). The expression levels of EMD were normalized to those of GAPDH.

### Western blotting

Proteins from self-beating embryoid bodies (EBs) were extracted with RIPA buffer (Nacalai Tesque, Japan). The proteins were loaded on Mini-PROTEAN TGX Gels (Bio-Rad, 4% to 15%) and then electrophoretically separated and transferred onto nitrocellulose blotting membranes (GE Healthcare). The primary antibodies were anti-emerin (Abcam), anti-SYNE1 (Millipore), and anti-GAPDH (Cell Signaling Technology). Protein expression was visualized with appropriate horseradish peroxidase-conjugated secondary antibodies (anti-mouse HRP 1:2000, anti-rabbit HRP 1:2000) and enhanced chemiluminescence (Amersham Biosciences, Piscataway, NJ, USA), and was detected using an LAS-3000 luminoimager (Fujifilm Techno Products, Ayase-shi, Kanagawa, Japan). Protein bands were quantified using the ImageJ software (NIH, Bethesda, MD, USA).

### Immunofluorescence

Colonies of undifferentiated iPS cells plated on a layer of mouse embryonic fibroblasts (feeder cells), iPS cell-derived cardiomyocytes, or NRVCs plated on fibronectin-coated dishes were fixed with 4% paraformaldehyde (MUTO Pure Chemicals, Tokyo, Japan) for 30 min at 4 °C. After that, the cells were permeabilized with 1% Triton X-100 and blocked with ImmunoBlock (DS Pharma Biomedical, Osaka, Japan). The samples were incubated at 4 °C overnight with each of the following primary antibodies: anti-OCT3/4 (Santa Cruz Biotechnology, CA, USA), anti-NANOG (Abcam, Camb, UK), anti-SSEA3 (Millipore, MA, USA), anti-SSEA4 (Millipore), anti-Tra1-81 (Millipore), anti-Tra1-60 (Millipore), anti-troponin T (Thermo Scientific, MA, USA), anti-α-actinin (Sigma-Aldrich), anti-NKX2.5 (Santa Cruz Biotechnology), anti-HCN4 (Abcam), anti-emerin (Abcam, Sanata Cruz Biotechnology), anti-LAMIN A/C (Santa Cruz Biotechnology), anti-SUN1 (Abcam), anti-LBR(Abcam), anti-NPC(Abcam) and anti-SYNE1 (ATLAS). Preparations were incubated for 1 hour at room temperature with the isotype-specific secondary antibodies: an Alexa Fluor 488-conjugated chicken anti-rabbit IgG antibody, Alexa Fluor 594-conjugated goat anti-mouse IgG antibody, Alexa Fluor 488-conjugated goat anti-rat IgM antibody, Alexa Fluor 594-conjugated goat anti-mouse IgM antibody, Alexa Fluor 488-conjugated goat anti-mouse IgG antibody, Alexa Fluor 594-conjugated chicken anti-goat IgG antibody, Alexa Fluor 633-conjugated goat anti-mouse IgG antibody and an Alexa Fluor 555-conjugated goat anti-rabbit IgG antibody, which were all acquired from Invitrogen. Nuclei were counterstained with 50 ng/mL 4′,6-diamidino-2-phenylindole (DAPI; Invitrogen) or 1 μg/mL Hoechst 33258 (Lonza). Fluorescent signals were detected under a fluorescence laser microscope (BZ9000 Keyence, Osaka, Japan) with ×10/0.45 numerical aparature (NA)or a confocal microscope with ×40/0.75 NA, ×63/1.2 NA or a ×63 × 3/1.2 NA oil-immersion objective (LSM 5 DUO Carl Zeiss, Jena, Germany).

### Measurement nuclear and cytoplasmic size of cardiomyocytes and assessment of nuclear invagination

The cytoplasm area was assessed by means of troponin T-positive area obtained by confocal microscopy (LSM5 DUO at ×40 × /0.75 NA), and the nuclear area was assessed by means of the DAPI-positive area set to the middle of a visual field under the LSM5 DUO at ×63 × 3/1.2 NA oil-immersion objective. Analysis of individual cells was performed in a blinded experiment using the area calculating system of LSM510. We performed three dimensional images with 0.35 μm z-stack thickness to assess nuclear invagination. Nuclear volume was assessed with DAPI staining by the IMARIS software (Bitplane Company, Zurich, Switzerland). Nuclear invagination was assessed with LAMIN A/C or LBR as previously described^26^ and with mag fluo4 as previously described^67^. Nuclear invagination positivity was defined as intrusion of LAMIN A/C or LBR inside the nucleoplasm (longer than 1 μm) on one slice and three dimensional images.

### Patients’ consent

All subjects provided informed consent for blood testing for genetic aberrations associated with EDMD. The Ethics Committee of Kanazawa University approved the isolation and use of patients’ and control somatic cells for iPS cell experiments (approval No. 1147), which were performed only after the patients and controls had provided written informed consent. Isolation and use of patients’ and control cells were approved by the Ethics Committee of Keio University (20-92-5) and were performed after written informed consent was obtained. The study was performed conforming to the Declaration of Helsinki.

### Preparation of human iPS cells

To study the role of emerin in human cardiomyocytes, we focused on the human disease that is associated with a mutation in EMD^33^. The mutation in EMD leads to X-linked EDMD. EDMD is characterized by muscle weakness, DCM, and cardiac conduction defects. Therefore, we planned to derive iPS cells from a patient with EDMD, differentiate them into cardiomyocytes, and characterize the EDMD-iPS cell-derived cardiomyocytes. The iPS cells were derived from T lymphocytes by means of a Sendai virus encoding OCT3/4, SOX2, KLF4, and MYC^58^,^68^. Briefly, peripheral blood mononuclear cells (PBMCs) were collected and transferred at 1.5 × 10^6^/well to a fresh anti-CD3 antibody-coated 6-well plate, and incubated for an additional 24 hours. Then, solutions containing the Sendai virus vectors individually carrying each of OCT3/4, SOX2, KLF4, and MYC were added at multiplicity of infection (MOI) of 10. After 24 hours of infection, the medium was changed to a fresh KBM 502 medium (KOHJIN BIO, Saitama, Japan), and the cells were collected and split at 5 × 10^4^ cells into 10-cm plates preseeded with mouse embryonic fibroblasts. After additional 24 hours of incubation, the medium was changed to the hiPSC medium (described below) supplemented with 4 ng/mL basic fibroblast growth factor (bFGF). Approximately 20 to 30 days after the infection, iPS cell colonies appeared and were picked. Sendai virus vectors carrying the same four specific transcriptional factors (separately) were purchased from Life Technologies (CytoTune-iPS Reprogramming Kit).

### Genome sequencing

All subjects provided informed consent for blood testing for genetic aberrations associated with EDMD. DNA sequencing was used to confirm the presence of the relevant mutation in patient-derived iPS cells. Genomic DNA was isolated using the Gentra Puregene Cell Kit (Qiagen, Venlo, Netherland), and the region encoding the mutation was amplified by PCR. The PCR product was analyzed by electrophoresis in a 1% agarose gel and purified using a Wizard SV Gel and PCR Clean-Up System (Promega, WI, USA). The purified PCR product was sequenced with the original primers shown in the Supplementary Table.

### Human iPS cell culture

Human iPS cells were maintained for several passages on irradiated mouse embryonic fibroblast feeder cells in the hiPSC culture medium: 80% of DMEM/F12 (Sigma-Aldrich, MO, USA), 20% of the Knockout Serum Replacement (Invitrogen, CA, USA), 4 ng/mL bFGF (WAKO, Osaka, Japan), 2 mmol/L L-glutamine (Invitrogen), 0.1 mmol/L nonessential amino acids (Sigma-Aldrich), 0.1 mmol/L 2-mercaptoethanol, 50 U/mL penicillin, and 50 mg/mL streptomycin (Invitrogen). The hiPSC medium was changed every 2 days, and the cells were passaged using 1 mg/mL collagenase IV (Invitrogen) every 5–7 days. After several passages, these cell lines were cultured in the human iPS cell medium consisting of Repro Stem (Repro cell) and 50 U penicillin and 50 mg/mL streptomycin (Invitrogen).

### *In vitro* cardiomyocyte differentiation

The iPSCs were harvested using 1 mg/mL collagenase IV, and transferred to ultra-low attachment plates (Corning, NY, USA) in the Repro stem medium supplemented with 10 ng/mL BMP4 (Wako) and 5 μmol/L blebbistatin (Sigma) from day 0 to day 1. The medium was changed to Stempro34 (Gibco) supplemented with 50 μg/mL ascorbic acid (Sigma), 2 mmol/L glutaMAX (Invitrogen), 10 ng/mL BMP4, and 25 ng/mL activin A from day 1 to day 3. On day 3, the medium was changed to Stempro34 supplemented with 50 μg/mL ascorbic acid and 2 mmol/L glutaMAX. On day 4.5, the medium was changed to Stempro34 supplemented with 50 μg/mL ascorbic acid, 2 mmol/L glutaMAX, and 2.5 μmol/L IWR-1 (Sigma). On day 8.5, the medium was changed to the differentiation medium consisting of α Minimum Essential Medium (Gibco) supplemented with 2 mmol/L L-glutamine (Invitrogen), 0.1 mmol/L nonessential amino acids (Sigma), 0.1 mmol/L 2-mercaptoethanol, 50 U/mL penicillin, and 50 mg/mL streptomycin (Invitrogen), and 10% of fetal bovine serum (Gibco). The medium was replaced every second day or every third day. After 20 to 30 days of differentiation, self-contracting EBs were picked and placed into fresh ultra-low attachment plates (Corning). For immunohistochemical and Ca^2+^ imaging analyses, a single-cell suspension of cardiomyocytes (isolated from spontaneously contracting EBs) by means of 0.25% trypsin (Invitrogen), 1 mg/mL type IV collagenase (Invitrogen), and ADS buffer (116 mmol/L NaCl, mmol/L KCl 5.4, 1 mmol/L NaH_2_PO_4_, 0.8 mmol/L MgSO_4_, 5.5 mmol/L glucose, 20 mmol/L HEPES, pH 7.35, adjusted with NaOH) for 30 min. at 37 °C. The cells were plated in fibronectin-coated dishes and incubated for 5 days in the presence of 20% FBS. To analyze the iPSC-derived cardiomyocytes, the mixtures of contracting EBs derived from two independent iPSC lines were used in each case.

### Immunohistological fluorescence assays

Histological sections of the autopsied heart from an 83-year-old patient with EDMD and a 78-year-old control patient were permeabilized with 1% Triton X-100 after deparaffinization, and the antigens were activated with Histo VT one (Nacalai Tesque) at 90 °C for 40 min. After that, cardiomyocyte-containing tissue slices were blocked with ImmunoBlock (DS Pharma Biomedical) at 4 °C overnight and were incubated at 4 °C overnight with each of the following primary antibodies: anti-LAMIN A/C (Santa Cruz Biotechnology) and anti-troponin T (Thermo Fisher Scientific, MA, USA). Preparations were incubated for 1 hour at room temperature with the isotype-specific secondary antibodies: an Alexa Fluor 488-conjugated chicken anti-rabbit IgG antibody and Alexa Fluor 594-conjugated goat anti-mouse IgG antibody, both obtained from Invitrogen. Nuclei were counterstained with 50 ng/mL DAPI (Invitrogen). Fluorescent signals were detected using a fluorescence laser microscope (BZ9000 Keyence, Osaka, Japan) or by confocal microscopy with a ×40/1.2 NA or ×63 × 3/1.2 NA oil-immersion objective (LSM 5 DUO Carl Zeiss, Jena, Germany) just as during the measurement of the cardiomyocyte cytoplasmic and nuclear area.

### Statistical analyses

Continuous data are presented as mean ± SD. The significance of differences between two means was evaluated by unpaired t tests. Fisher’s exact probability test was used for comparisons between groups. Comparisons between more than two groups involved analysis of variance (ANOVA) followed by Dunnett’s test. Categorical data are presented as mean ± SE and are compared by using Chi-squared test. All analyses were conducted in EZR^69^; P < 0.05 was assumed to denote statistical significance.

## Additional Information

**How to cite this article**: Shimojima, M. *et al*. Emerin plays a crucial role in nuclear invagination and in the nuclear calcium transient. *Sci. Rep.* **7**, 44312; doi: 10.1038/srep44312 (2017).

**Publisher's note:** Springer Nature remains neutral with regard to jurisdictional claims in published maps and institutional affiliations.

## Supplementary Material

Supplementary Information
