# Supplementary material for: Emerin plays a crucial role in nuclear invagination and in the nuclear calcium transient
Source: Sci Rep. 2017 Mar 14;7:44312. doi: 10.1038/srep44312 (PMC5349585; doi:10.1038/srep44312)
Supplement: Supplementary Information [file srep44312-s1.pdf]

Supplementary Material

Emerin plays a crucial role in nuclear invagination and in the nuclear calcium transient

Short title: Emerin regulates nuclear calcium dynamics

Masaya Shimojima[1,2], *Shinsuke Yuasa[1], Chikaaki Motoda[1], Gakuto Yozu[1], Toshihiro Nagai[3], Shogo Ito[1], Mark Lachmann[1], Shin Kashimura[1], Makoto Takei[1], Dai Kusumoto[1], Akira Kunitomi[1], Nozomi Hayashiji[1], Tomohisa Seki[1], Shugo Tohyama[1], Hisayuki Hashimoto[1], Masaki Kodaira[1], Toru Egashira[1], Kenshi Hayashi[2], Chiaki Nakanishi[1,2], Kenji Sakata[2], Masakazu Yamagishi[2], Keiichi Fukuda[1]

1. Department of Cardiology, Keio University School of Medicine, 35 Shinanomachi Shinjuku-ku, Tokyo 160-8582, Japan.
2. Division of Cardiovascular Medicine, Kanazawa University Graduate School of Medicine, Takara-machi 13-1, Kanazawa, Ishikawa 920-8640, Japan.
3. Electron Microscope Laboratory, Keio University School of Medicine, Tokyo 160-8582, Japan.

Corresponding author: Shinsuke Yuasa, M.D, Ph.D., Assistant Professor, Department of Cardiology, Keio University School of Medicine, 35 Shinanomachi Shinjuku-ku, Tokyo, 160-8582, Japan, Tel.: +81 3-5363-3373, Fax: +81 3-5363-3875, E-Mail: yuasa@keio.jp

Supplementary Figure 1. Effects of hypertrophic stimuli on neonatal rat ventricular cardiomyocytes (NRVCs)

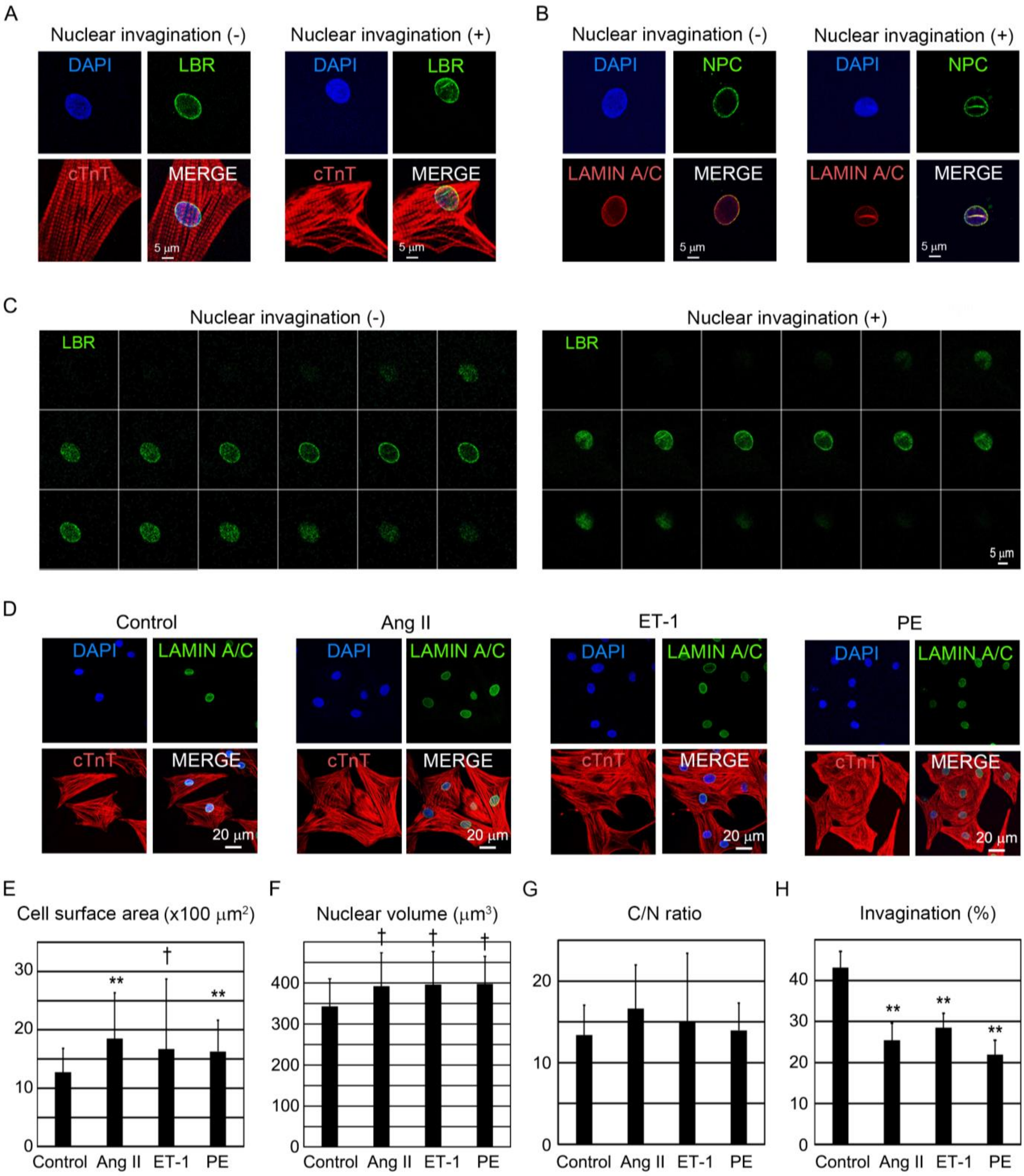

A. Confocal microscopic images of immunostaining for cTnT (red), LBR which is an inner nuclear membrane protein (green), and nuclei (blue) in NRVCs. The scale bar is 5 µm. Top: a cardiomyocyte without nuclear invagination. Bottom: a cardiomyocyte with nuclear invagination. B. Confocal microscopic images of immunostaining for cTnT (red), NPC which transverses nuclear membrane (green), and nuclei (blue) in NRVCs. The scale bar is 5 µm. Top: a cardiomyocyte without nuclear invagination. Right: a cardiomyocyte with nuclear invagination. C. Confocal microscopic images of immunostaining for cTnT (red), LBR (green), and nuclei (blue) in NRVCs. The scale bar is 5 µm. Left: a cardiomyocyte without nuclear invagination. Right: a cardiomyocyte with nuclear invagination. C. Confocal microscopic images of immunostaining for LBR (green) with 3D reconstruction serial images, in NRVCs. The scale bar is 5 µm. D. Confocal microscopic images of immunostaining for cTnT (red), LAMINA/C (green), and nuclei (blue) in NRVCs. The scale bar is 20 µm. E. Bar graphs showing cell surface area in NRVCs exposed to vehicle (control; n = 100), Ang II (n = 26), ET-1 (n = 108), or PE (n = 79) for 48 hours in culture. The data are mean ± SD. F. Bar graph showing nuclear volume in NRVCs exposed to vehicle (control; n = 162), Ang II (n = 110), ET-1 (n = 165), or PE (n = 137) for 48 hours in culture. The data are mean ± SD. G. Bar graphs showing the ratio of cell surface area to nuclear area in NRVCs exposed to vehicle (control; n = 100), Ang II (n = 26), ET-1 (n = 108), or PE (n = 79) for 48 hours in culture. The data are mean ± SD. H. Bar graphs showing the percentage of nuclear invagination assessed with 3D construction exposed to vehicle (control; n = 162), Ang II (n = 110), ET-1 (n = 165), or PE (n = 137) for 48 hours in culture. The data are mean ± SE . **P* < 0.05 compared to the control. ***P* < 0.01 compared to the control. †P < 0.001 compared to the control.

Supplementary Figure 2. Nuclear calcium transients in neonatal rat ventricular cardiomyocytes (NRVCs) exposed to hypertrophic stimuli

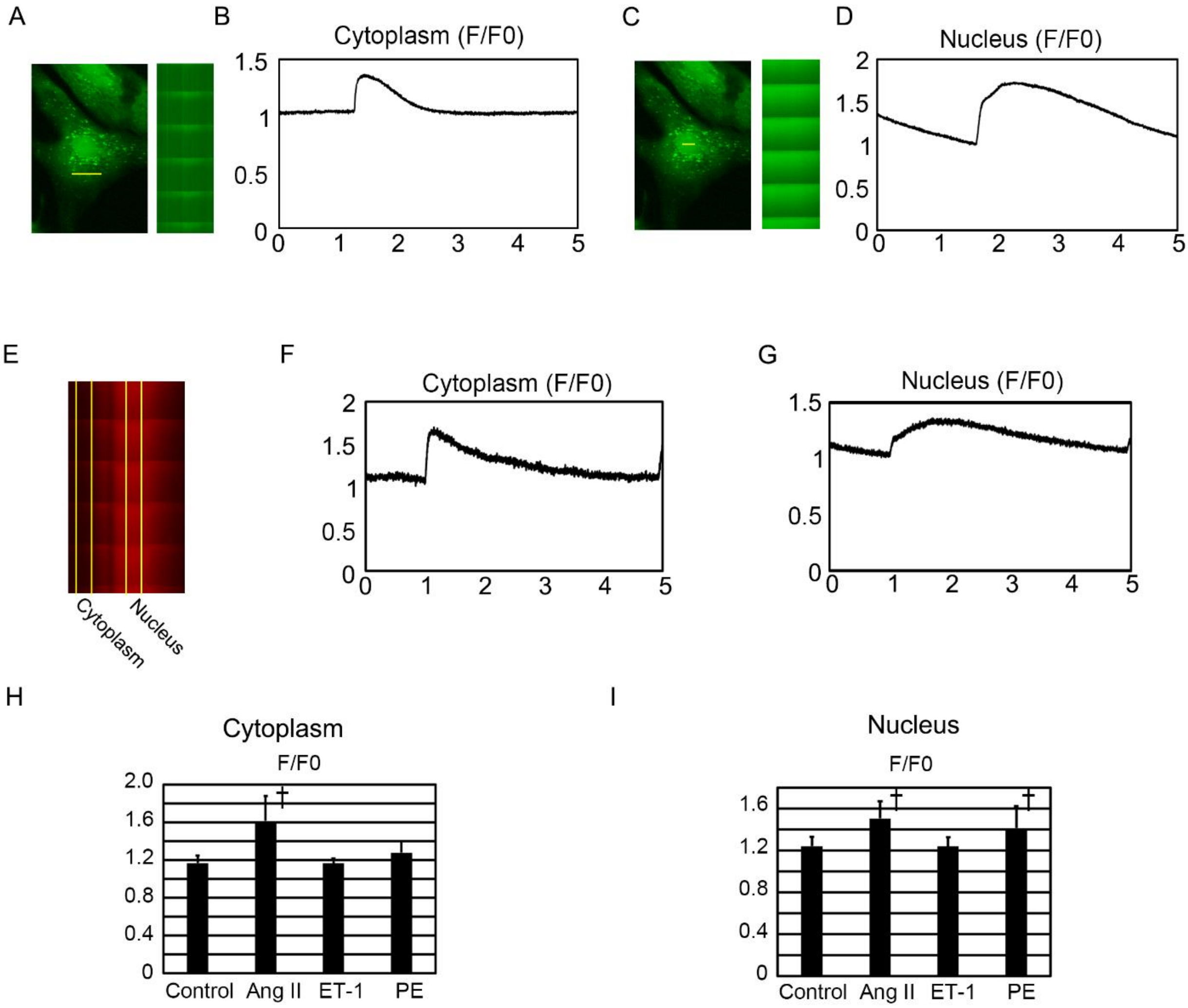

A. Original recording of only cytoplasmic Ca^{2+} transients in NRVC with fluo4. B. Line scan imaging of only cytoplasmic Ca^{2+} transients in cardiomyocytes with fluo4. C. Original recording of only nucleus Ca^{2+} transients in cardiomyocytes with fluo4. D. Line scan imaging of cytoplasmic Ca^{2+} transients in cardiomyocytes with fluo4. E. Original recording of cytoplasmic and nucleus Ca^{2+} transients in NRVC with a different Ca indicator, Rhod4. F. Line scan imaging of only cytoplasmic Ca^{2+} transients in cardiomyocytes with Rhod4. G. Line scan imaging of nuclear Ca^{2+} transients in cardiomyocytes with Rhod4. H. Average values of the effects of hypertrophic stimuli on time to F/F0 in the cytoplasm of NRVCs exposed to vehicle (control; n = 19), Ang II (n = 42), ET-1 (n = 21), or PE (n = 29). I. Average values of the effects by hypertrophic stimuli on time to F/F0 in the nucleus of NRVCs exposed to vehicle (control; n = 19), Ang II (n = 42), ET-1 (n = 21), or PE (n = 29). **P* < 0.05 compared to the control. ***P* < 0.01 compared to the control. †P < 0.001 compared to the control.

Supplementary Figure 3. Characterization of *Emd* knockdown neonatal rat ventricular cardiomyocytes (NRVCs)

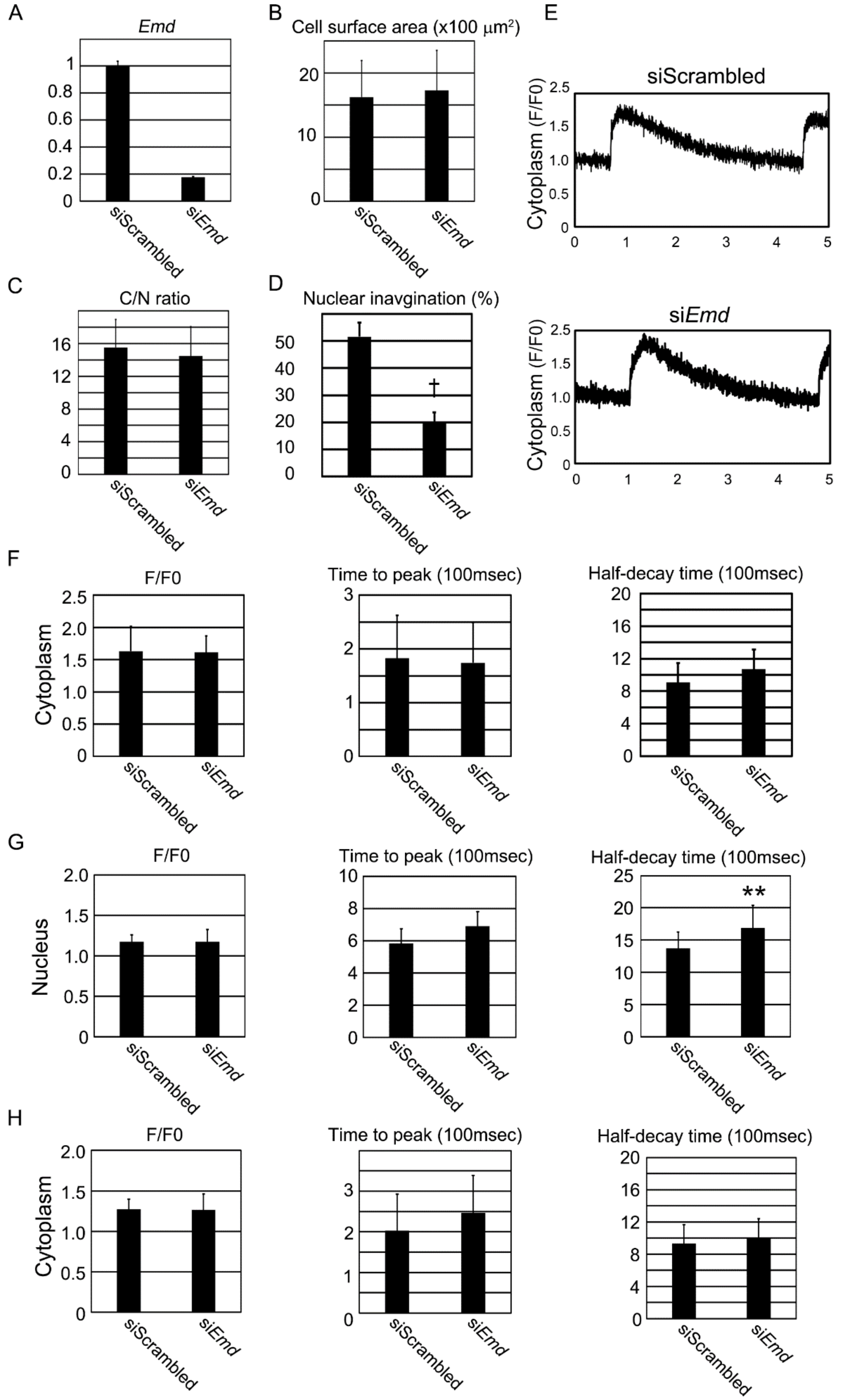

A. Quantitative RT-PCR analysis of *Emd* in NRVCs transfected with control scrambled siRNA (siScrambled, left) or siRNA for *Emd* (si*Emd*, right) (n = 3). The data are mean ± SD. B. Bar graphs showing cell surface area in NRVCs transfected with siScrambled (n = 69) or si*Emd* (n = 47) for 48 hours in culture. The data are mean ± SD. C. Bar graphs showing the ratio of cell surface area and nuclear area in NRVCs transfected with siScrambled (n = 69) or si*Emd* (n = 47). The data are mean ± SD. D. Bar graphs showing the percentage of incidence of NRVCs with nuclear invagination assessed with 3D construction, exposed to siScrambled (n = 95) or si*Emd* (n = 138). The data are mean ± SE. E. Original recordings of the cytoplasmic Ca^{2+} transient of NRVCs transfected with siScrambled (n = 25) or si*Emd* (n = 29).F. Average values of the effects of the *Emd* knockdown on F/F0, time to peak, and half-decay time in the cytosol of NRVCs with fluo4.G. Average values of the effects of the *Emd* knockdown on F/F0, time to peak, and half-decay time in the nucleus of NRVCs with Rhod4. H. Average values of the effects of the *Emd* knockdown on F/F0, time to peak, and half-decay time in the cytosol of NRVCs with Rhod4. **P* < 0.05 compared to the control. ***P* < 0.01 compared to the control. †P < 0.001 compared to the control.

Supplementary Figure 4. Characterization of cytosolic and nuclear calcium transients in *Emd* knockdown neonatal rat ventricular cardiomyocytes (NRVCs) subjected to hypertrophic stimuli

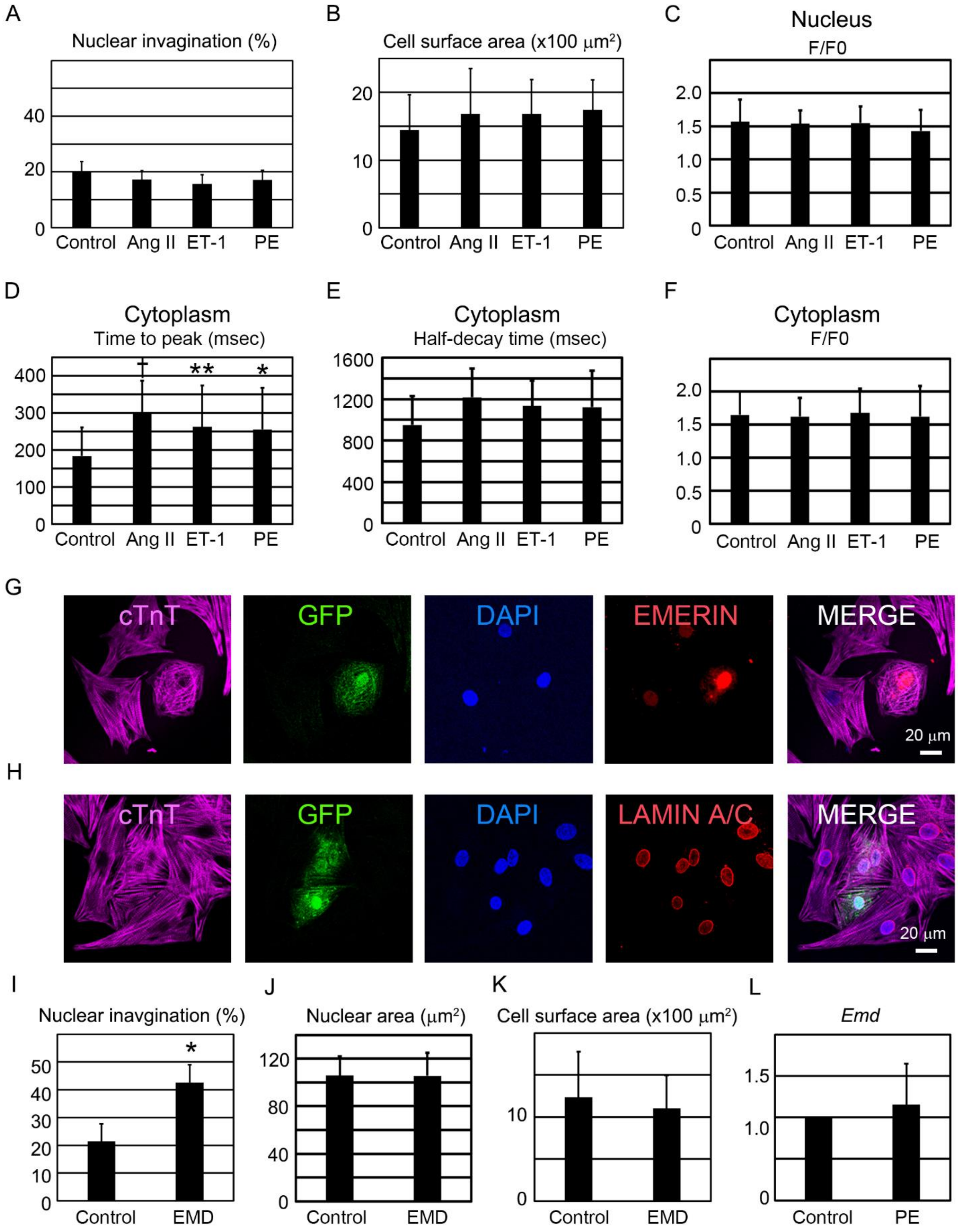

A. Bar graphs showing the percentage of nuclear invagination assessed with 3D construction exposed to transfected with siRNA against *Emd* after exposure to vehicle (control; n =138), Ang II (n =134), ET-1 (n = 115), or PE (n =117). The data are mean ± SE. B. Bar graphs showing cell surface area in NRVCs with siRNA against *Emd* exposed to vehicle (control; n = 62), Ang II (n = 47), ET-1 (n = 56), or PE (n = 55) for 48 hours in culture. The data are mean ± SD. C. Average values of the effects of hypertrophic stimuli on F/F0 in the nucleus of NRVCs transfected with siRNA against *Emd* after exposure to vehicle (control; n = 29), Ang II (n = 25), ET-1 (n = 49), or PE (n = 24). D,E,F. Average values of the effects of hypertrophic stimuli on time to peak, half-decay time, and F/F0 in the cytosol of NRVCs transfected with siRNA against *Emd* after exposure to vehicle (control; n = 29), Ang II (n = 25), ET-1 (n = 49), or PE (n = 24). G. Confocal microscopic images of immunostaining for cTnT (pink), GFP (green), EMERIN (red) and nuclei (blue) in NRVCs with overexpression of both GFP and EMERIN. The scale bar is 20 µm. H. Confocal microscopic images of immunostaining for cTnT (pink), GFP (green), LAMIN A/C (red) and nuclei (blue) in NRVCs with overexpression of both GFP and EMERIN. The scale bar is 20 µm. I. Bar graphs showing the percentage of incidence of NRVCs with nuclear invagination transfected with GFP (n = 42) or GFP-IRES-EMERIN (n = 61). The data are mean ± SE. J, K. Bar graphs showing the nuclear area(H) and cell surface area(I) transfected with GFP (n =42) or GFP-EMERIN (n =61). The data are mean ± SD. L. Quantitative RT-PCR analysis of *Emd* in NRVCs stimulated with PE for 48 hours (right) (n = 3). The data are mean ± SD.

Supplementary Figure 5. Characterization of EDMD-iPS cell-derived cardiomyocytes

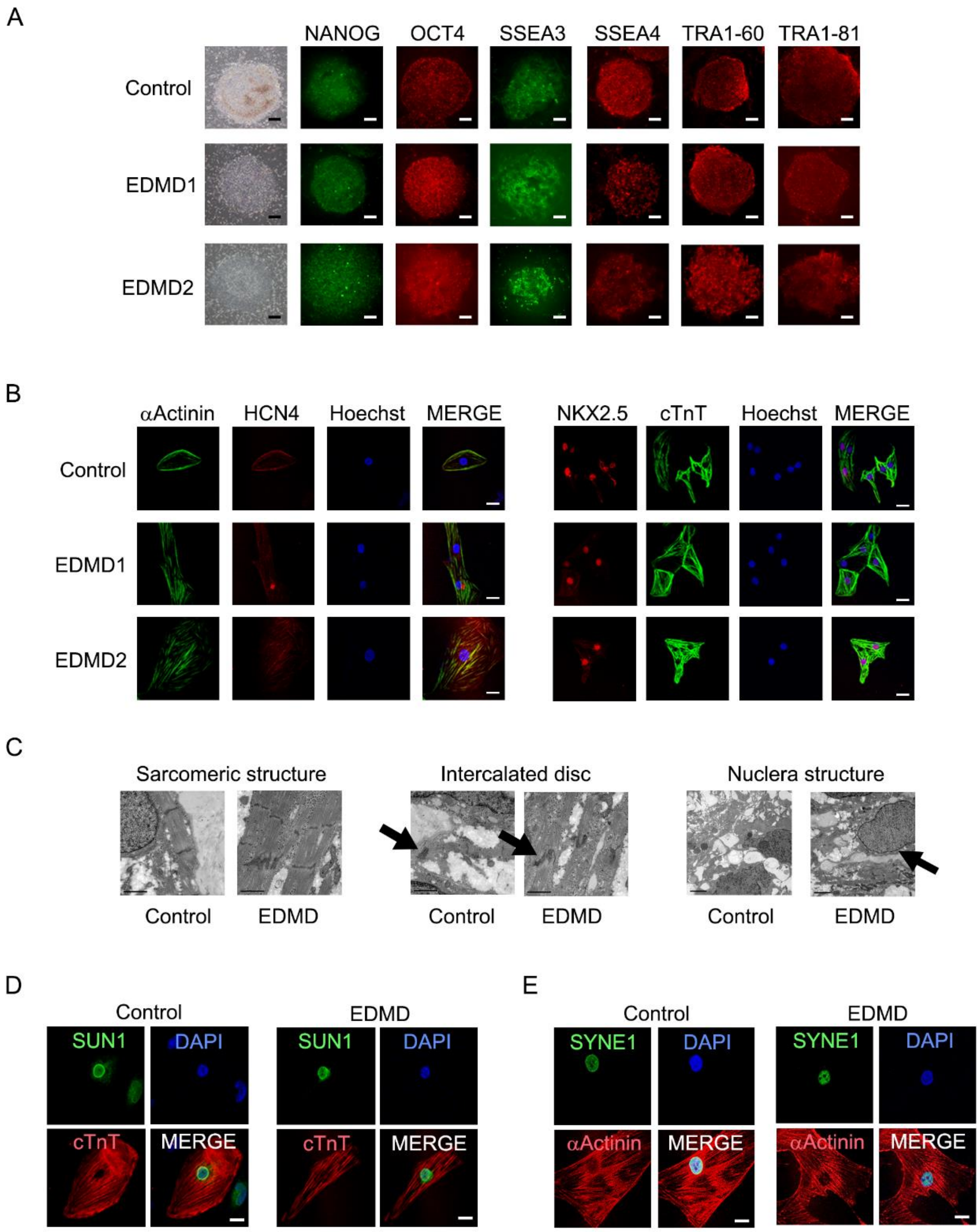

A. Immunofluorescent staining for stem cell markers (NANOG, OCT4, SSEA3, SSEA4, TRA1-60, and TRA1-81) in control iPS cells and two independent iPS cell clones of the EDMD case. The scale bar is 100 μm. B. Immunofluorescent staining for cardiomyocyte markers (α-actinin, HCN4, NKX2.5, and cardiac troponin T [cTnT]) in control and EDMD-iPS cell-derived cardiomyocytes. Nuclei were counterstained with DAPI. C. Electron microscopic images of control and EDMD-iPS cell-derived cardiomyocytes. The scale bar is 20 μm. D. Immunofluorescence staining for nuclear membrane markers (SUN1 and SYNE1) and cardiomyocyte markers (cTnT and α-Actinin) in control and EDMD-iPS cell-derived cardiomyocytes. Nuclei were counterstained with DAPI. The scale bar is 20 μm.

Supplementary Table

Primers for quantitative RT-PCR analysis of neonatal rat ventricular cardiomyocytes

Emd Forward	ggaggacgccttactttacca
Emd Reverse	ccatagatggggcgttccc
GAPDH Forward	tgggaagctggtcatcaac
GAPDH Reverse	gcatcaccccatttgatgtt

Primers for genomic DNA sequencing of the *EMD* gene

EMD Forward Exon6	gctcctggcccacttgctcc
EMD Reverse Exon6	ctaaggcagtcagccaggac
